# Efficacy and Interindividual Variability in Motor-Cortex Plasticity following Anodal tDCS and Paired-Associative Stimulation

**DOI:** 10.1155/2015/530423

**Published:** 2015-03-17

**Authors:** Wolfgang Strube, Tilmann Bunse, Berend Malchow, Alkomiet Hasan

**Affiliations:** Department of Psychiatry and Psychotherapy, Ludwig Maximilian University, 80336 Munich, Germany

## Abstract

Interindividual response variability to various motor-cortex stimulation protocols has been recently reported. Comparative data of stimulation protocols with different modes of action is lacking. We aimed to compare the efficacy and response variability of two LTP-inducing stimulation protocols in the human motor cortex: anodal transcranial direct current stimulation (a-tDCS) and paired-associative stimulation (PAS25). In two experiments 30 subjects received 1mA a-tDCS and PAS25. Data analysis focused on motor-cortex excitability change and response defined as increase in MEP applying different cut-offs. Furthermore, the predictive pattern of baseline characteristics was explored. Both protocols induced a significant increase in motor-cortical excitability. In the PAS25 experiments the likelihood to develop a MEP response was higher compared to a-tDCS, whereas for intracortical facilitation (ICF) the likelihood for a response was higher in the a-tDCS experiments. Baseline ICF (12 ms) correlated positively with an increase in MEPs only following a-tDCS and responders had significantly higher ICF baseline values. Contrary to recent studies, we showed significant group-level efficacy following both stimulation protocols confirming older studies. However, we also observed a remarkable amount of nonresponders. Our findings highlight the need to define sufficient physiological read-outs for a given plasticity protocol and to develop predictive markers for targeted stimulation.

## 1. Introduction

Noninvasive brain stimulation (NIBS) finds increasingly more applications in clinical neuroscience. Under the term NIBS, different techniques are summarized that allow a noninvasive stimulation of the brain but that are characterized by different modes of action [1]. Recent studies in the field have made clear that the response capacity to any NIBS protocol is subject of a significant interindividual variability [2–5]. Contrary to initial expectations, these studies displayed that a specified number of subjects will not show the expected effects following a given NIBS protocol but showed that these subjects may show no responses or even the opposite effects. Taking into consideration that NIBS are currently been applied in clinical practice to treat various neuropsychiatric disorders (e.g., depression, schizophrenia, and stroke), it is necessary to better understand the response variability following NIBS. In particular studies comparing the efficacy of different NIBS techniques are lacking. Thus, we decided to perform a comparative study of the efficacy and the response variability of two well-established NIBS protocols with different physiological modes of action: transcranial direct current stimulation (tDCS) and paired-associative stimulation (PAS).

tDCS is one of the most widespread techniques in clinical trials and experimental settings, because the application is simple and the neurophysiological and behavioural effects are discussed to be rather strong. Animal studies showed that tDCS modulates spontaneous neuron activity by a tonic depolarisation (anodal tDCS) or hyperpolarisation (cathodal tDCS) of their membrane potentials resulting in consecutive long-lasting changes in the neuronal firing rates [6–8]. A more recent animal study conducted on motor-cortex slices from the mouse brain slices confirmed the polarity-specific effects of anodal tDCS and showed that anodal tDCS induces long-lasting synaptic potentiation outlasting the duration of stimulation [9]. Human studies confirmed these findings by showing similar polarity-dependent changes in motor-cortical excitability following anodal or cathodal tDCS [7]. Anodal tDCS led to an increase of motor-evoked potentials (MEPs) and cathodal tDCS resulted in a decrease of MEPs following a stimulation of 13 minutes or respective 9 minutes [10, 11]. Pharmacological challenges in healthy subject demonstrated that this modulation in motor-cortical excitability following tDCS is critically dependent on calcium homeostasis and the activity of glutamatergic NMDA receptors [12, 13]. In summary, these findings from animal and human studies suggest that the after-effects following tDCS are related to neural plasticity and to the molecular mechanisms of long-term potentiation and long-term depression [7].

On the other hand, PAS is mainly used in experimental settings because the application is more complicated than the application of tDCS. Compared to all other NIBS, PAS has the strongest foundation in basic research [1]. PAS fulfils several criteria of a plasticity protocol, as the after-effects are long-lasting, persist the duration of the intervention, and are input-specific [14] and NMDA-dependent [15]. However, the special characteristics are that the after-effects of PAS seem to be synapse-specific following Hebbian principles and that they are related to spike-dependent plasticity [3, 14, 16]. Human studies indicate that the repeated pairing of an electric stimulus of a peripheral nerve (somatosensory afferent) followed by a single magnetic pulse of the contralateral motor cortex with an interstimulus interval of 25 ms (or adjusted to the individual N20-latency plus 2 ms) results in a long-lasting MEP increase in terms of LTP-like plasticity [1, 14, 16]. An adjustment of the interstimulus interval to 10 ms (or adjustment to the N20-latency minus 5 ms) causes a reduction of MEP amplitudes (LTD-like plasticity) [1, 14, 16].

The aim of this study was to compare the efficacy and response variability of two LTP-like plasticity inducing NIBS protocols using the most established stimulation configurations. Anodal tDCS was applied with 1 mA for 13 minutes [7, 17] and PAS25 consisted of 180 pairs of peripheral nerve stimulation followed by a magnetic pulse with an interstimulus interval of 25 ms [14, 15, 18]. Only one previous study has yet directly compared these two LTP-like plasticity inducing protocols [5]. This study showed the same level of excitability change and the same proportion of responders following anodal tDCS and PAS25 but no mean change of motor-cortex excitability when all subjects were analysed [5]. These findings contrast earlier reports that investigated either anodal tDCS (for review see [7]) or PAS (for review see [16]) and showed significant group-level changes in cortical excitability. We aimed to either replicate or refute these initial findings and to extend the current knowledge of response variability following NIBS. We hypothesised that both plasticity protocols will result in a mean increase in cortical excitability but that a certain proportion of subjects will not show the expected results.

## 2. Methods

### 2.1. Participants

The study protocol was conducted in accordance with the Declaration of Helsinki and approved by the Ethics Committee of the Ludwig Maximilians University of Munich. After giving written informed consent, 30 healthy participants, all aged between 19 and 42 years, were included in the study. All participants underwent a standardized biographic interview and testing of hand preference [19]. Participants with contraindication to TMS, peripheral nerve stimulation, or tDCS were excluded. All subjects were medication-free and screened by clinically experienced psychiatrists for psychiatric comorbidities. Sociodemographic variables are presented in Table 1.

### 2.2. Study Design

Subject received two experimental sessions (anodal tDCS versus PAS25) on two different days and sessions for each subject were 7 to 8 days apart. Subjects received on the first study day anodal tDCS and on the second study day PAS25. The sociodemographic interview was performed on the first study day. All experiments were performed by the same investigator (Wolfgang Strube).

### 2.3. TMS Procedure and Cortical Excitability

Subjects were examined in half-reclined sitting position with their arms resting passively supported. Electromyographic activity (EMG) was recorded by surface electrodes on the right first dorsal interosseous muscle (FDI). Raw signals were amplified and bandpass-filtered (3 Hz–2 kHz range) using a Digitimer D-360 amplifier setup (Digitimer Ltd., UK). Recordings were digitized using a 1401 data acquisition interface (Cambridge Electronic Design Ltd., Cambridge, UK) controlled by Signal Software (Version 5, Cambridge Electronic design, Cambridge, UK). Each recording was manually analyzed offline to exclude movement-artefact related aberrant data. Motor-evoked potentials (MEP) were induced by TMS applied to the left primary motor cortex (M1) with a standard figure-of-eight magnetic coil (outer diameter 70 mm, The Magstim Company Ltd., UK) and a monophasic Magstim Bistim^2^ stimulator (The Magstim Company Ltd., UK). Throughout all experiments, the coil was held tangentially to the skull, with the handle pointing backwards and in a 45° angle lateral to the midline. The stimulation site that produced the largest motor-evoked potential (MEP) at moderately suprathreshold stimulation intensities was defined as the hot spot and marked for further optimal coil positioning.

**Figure 1:**
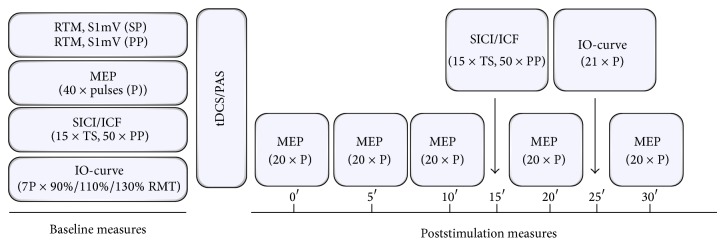
Experimental course and design. At baseline RMT and S1mV were recorded for single-pulse (SP) and paired-pulse (PP) TMS-measures. Then, 40 MEP with test stimulus intensity pulses (P), SICI and ICF (15 test stimuli and 10 paired stimuli for each ISI), and IO-curves were obtained followed by either anodal tDCS or PAS. After stimulation 20 MEP were recorded at five different time points for 30 minutes with SICI/ICF and IO-curves measured at 15 and 25 minutes.

RMT was recorded in the resting FDI muscle and defined as the minimum stimulator intensity that resulted in an MEP amplitude of ≥50 *µ*V in at least 5 of 10 measurements. The stimulation intensity corresponding to MEP amplitudes of 1 mV (±0.3 mV) (S1mV) was adjusted at baseline and kept unchanged throughout the experiments. Single-pulse MEP measurements using the S1mV intensity were conducted at baseline (40 stimuli) and after stimulation (time points 0, 5, 10, 20, and 30 minutes; 20 stimuli at each time point) to monitor after-effects following both plasticity protocols (PAS and tDCS). To test for after-effects on cortical recruitment, input-output curves (IO) were measured at baseline and 8 minutes after stimulation using an increasing stimulus intensity order (90%, 110%, and 130% of RMT) with 7 stimuli for each intensity (Figure 1). Single- and paired-pulse TMS was applied at 0.2 Hz.

To assess the after-effects of the respective stimulation types on inhibitory and facilitatory intracortical networks, short-latency intracortical inhibition (SICI) and intracortical facilitation (ICF) were obtained at baseline and 15 minutes after stimulation using a standardized paired-pulse protocol [20]. The conditioning stimulus was set at 80% RMT intensity and the test stimulus at S1mV (±0.3 mV) in the resting FDI. The intensity of the test pulse was not adjusted after the intervention for paired-pulse measures. In total, 65 randomised stimuli were applied, with 15 stimuli using the test stimulus alone and 10 stimuli for each interstimulus interval (ISI) (SICI: 2 ms and 3 ms; ICF: 7 ms, 9 ms, and 12 ms).

### 2.4. Anodal tDCS

Anodal tDCS was applied through saline soaked rectangular surface sponge-electrodes (35 cm²) using a CE-certified standard stimulator (DC-Stimulator-Plus, NeuroConn GmbH, Ilmenau, Germany). The anodal electrode was positioned on the left side of the skull over the representational field of the right FDI as identified by TMS; the cathodal electrode was contralaterally above the right orbit. The stimulation intensity of the tonic electrical field was set at 1 mA and applied for a total duration of 13 minutes [7, 17], which has been consistently shown in previous tDCS publications to be an optimal duration time for the induction of cortical excitability changes in terms of LTP-like plasticity lasting for approximately one hour following tDCS [7].

### 2.5. PAS25

According to foregoing publications [14, 15, 18], the PAS25 protocol consisted of 180 pairs of peripheral nerve stimuli followed by TMS stimuli after an interstimulus interval (ISI) of 25 ms. The peripheral nerve stimulation was applied to the right ulnaris nerve at the level of the wrist using a CE-certified DS7A peripheral nerve stimulator (Digitimer Ltd., UK). The stimulation intensity was set at 300% of the individual perceptual threshold, resulting in an average electrical intensity of 9.8 ± 2.1 mA, which has been demonstrated to result in a reliable plasticity response [21]. The TMS stimuli were applied to the motor-cortical representation of the right FDI as identified in the excitability measurements. To maintain a constant level of attention during the stimulation, subjects were asked to watch their right hand, silently count the number of stimuli delivered, and report the adding number to the examiners request every 20–30 stimuli (random choice by examiner). All subjects mean count of the total number of paired stimuli was 176–182 (mean = 179.5 ± 1.5) and did not significantly differ from the total count of 180 stimuli, indicating a sufficient level of attention [22, 23].

## 3. Statistics

For statistical analysis, SPSS 22 for Windows was used and the level of significance was set at alpha = 0.05. To test for differences concerning baseline parameters between the two experimental sessions paired-samples *t*-tests were computed for all depending variables. Cortical excitability changes were expressed as increase or decrease in mean MEP amplitudes before and after stimulation. As the assumption of normal data distribution was violated for most depending variables (Kolmogorov-Smirnov test, *P* between <0.001 and 0.043), square root transformations were applied to meet the requirements to conduct RM-ANOVAs. To test the time course of plasticity changes, a RM-ANOVA (6 × 2) with the factors “time course” (baseline, 0 min, 5 min, 10 min, 20 min, and 30 min) and “stimulation type” (anodal tDCS and PAS) and another RM-ANOVA (2 × 2) with the factors time (baseline, mean post-MEPs averaged) and again “stimulation type” (anodal tDCS and PAS) were performed. In the next step, separate RM-ANOVAs for each stimulation type alone were conducted. To test for differences in the cortical recruitment, a RM-ANOVA with the factors “time course” (before and after stimulation) and intensity (90%, 110%, and 130% RMT) was conducted for both stimulation conditions separately. Changes in intracortical excitability over time were analysed with RM-ANOVAs with the factors “ISI” (test pulse, 2 ms, 3 ms, 7 ms, 9 ms, and 12 ms) and “time” (baseline, 15 minutes after stimulation). The same analyses were repeated with mean SICI and ICF values. When appropriate, that is, significant interactions in the RM-ANOVAs, Student's *t*-tests (paired, two-tailed) were performed to determine more specifically whether MEP amplitudes differed before and after plasticity induction within and between conditions. In cases of lacking interactions, no further *t*-tests were conducted. In the linear models, sphericity was tested with Mauchly's test and, if necessary (Mauchly's test < 0.05), the Greenhouse-Geisser correction was used. To test the individual response pattern, subjects were categorised in responders (R) and nonresponders (NR) to the respective LTP-protocol. To test for whether any of the obtained baseline parameters of cortical excitability showed a correlation with the excitability changes following anodal tDCS or PAS (relative mean post-MEPs), Pearson's correlation coefficients were applied. Additional analyses are described in Section 4. Data in tables are presented as mean ± standard deviation. In all figures, error bars refer to the standard error and graphs show untransformed data. Tables also show untransformed data.

## 4. Results

### 4.1. Descriptive Statistics

Subjects were aged between 19 and 42 years (mean 27.4 ± 4.8), 14 were female (47%), with one exception all were right handed (*n* = 29, 94%), the average body-height was 176.5 ± 9 cm, and 13 were smokers (43%) with a mean Fagerstrom-score of 3 (Table 1).

### 4.2. Baseline Differences

To compare baseline values in both experiments, paired-samples *t*-tests were computed for all depending variables: RMT, S1mV (both single- and double-pulse), 1 mV MEP, SICI (2 ms, 3 ms, mean), ICF (7 ms, 9 ms, and 12 ms, mean 9–12 ms), and recruitment curve (90%, 110%, and 130% RMT). None of the tested variables showed significant differences between the first and the second experimental sessions (all *P* > 0.129, Table 2).

### 4.3. Excitability Changes over Time

A repeated-measures analysis of variance (RM-ANOVA) was conducted with the factors “time course” (baseline, 0 min, 5 min, 10 min, 20 min, and 30 min) and “stimulation” (anodal tDCS, PAS). This analysis revealed a significant main effect on “time course” (*F*_5,25_ = 4.412, *P* = 0.001) but neither an effect on “stimulation” (*F*_1,29_ = 2.190, *P* = 0.150) nor an effect on the “time course × stimulation” interaction (*F*_5,25_ = 0.617, *P* = 0.687). In addition, the overall RM-ANOVA with the factors “time” (baseline, mean post-MEPs averaged) and again “stimulation” (anodal tDCS, PAS) showed also a significant main effect on “time” (*F*_1,29_ = 12.392, *P* = 0.001) and no effect on “stimulation” (*F*_1,29_ = 1.392, *P* = 0.248) or a “time × stimulation” interaction (*F*_1,29_ = 2.060, *P* = 0.162).

RM-ANOVAs separately computed for both stimulation protocols separately showed a significant main effect on “time course” in the PAS-group (*F*_5,25_ = 3.963, *P* = 0.002) but not in the tDCS-group (*F*_5,25_ = 1.408, *P* = 0.225). To analyse the general excitability changes following both stimulation types, a mean value of all poststimulation time points was included into an additional RM-ANOVA analysis, which showed a significant main effect on “time” for both anodal tDCS (*F*_1,29_ = 4.534, *P* = 0.042) and PAS (*F*_1,29_ = 16.041, *P* < 0.001).

**Figure 2:**
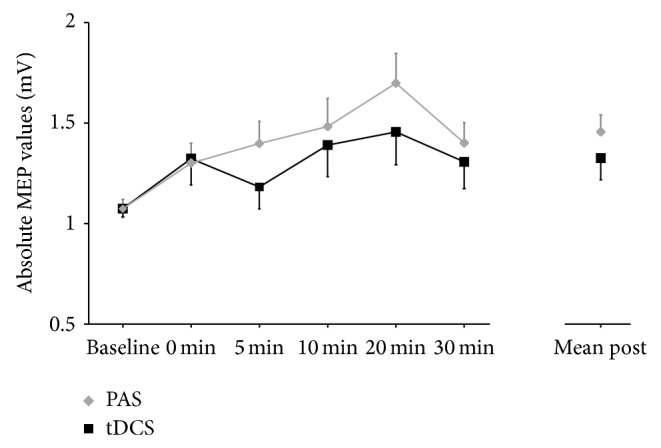
MEP values at baseline and all time points following anodal tDCS and PAS. MEP values are shown as untransformed values and scaled in mV and error bars representing the standard error of the mean.

For anodal tDCS, paired-samples *t*-tests showed a significant difference comparing baseline to the mean of all time points following stimulation (*t*_1,29_ = 2.13, *P* = 0.042). In the case of PAS, a significant increase in MEP size was found comparing baseline to the mean of all time points following stimulation (*t*_1,29_ = 4.01, *P* < 0.001) and at all single time points after stimulation (all *t*_1,29_ > 2.13, all *P* < 0.042) with the exception of 0 minutes after stimulation (*t*_1,29_ = 1.95, *P* = 0.061). Baseline MEPs did not differ between the anodal and the PAS conditions (*t*_1,29_ = 0.06, *P* = 0.953) and also mean post-MEPs did not differ between the anodal and the PAS conditions (*t*_1,29_ = 1.575, *P* = 0.126) (Figure 2).

To further explore the observed differences between the MEP increase in tDCS and PAS, we further conducted a RM-ANOVA of the standard deviations. This analysis revealed a trend-level effect on “time course” (*F*_5,25_ = 1.91, *P* = 0.097) and no effect on “stimulation” (*F*_1,29_ = 0.74, *P* = 0.397) and no “time course × stimulation” interaction (*F*_5,25_ = 1.02, *P* = 0.407). This finding can be explained by higher standard deviations after stimulation in both conditions.

A RM-ANOVA for the IO-curves with the factors “time” (baseline, after stimulation) and “intensity” (90%, 110%, and 130% RMT) revealed a significant main effect on “intensity” for both anodal tDCS (*F*_2,28_ = 179.87, *P* < 0.001) and PAS (*F*_2,28_ = 112.60, *P* < 0.001) but no effect on “time” (tDCS: *F*_1,29_ = 2.60, *P* = 0.118; PAS: *F*_1,29_ = 3.07, *P* = 0.090) and no “time × intensity” interaction (tDCS: *F*_2,28_ = 0.679, *P* = 0.515; PAS: *F*_2,28_ = 2.02, *P* = 0.151).

### 4.4. Paired-Pulse Measurements

For paired-pulse measurements, two additional RM-ANOVAs were conducted for both anodal tDCS and PAS separately with the factors “time” (baseline, 15 min after stimulation) and all “ISI” (test pulse, 2 ms, 3 ms, 7 ms, 9 ms, and 12 ms) or “mean ISI” (test pulse, mean SICI (2 ms, 3 ms), and mean ICF (9 ms, 12 ms)). In the case of anodal tDCS, the 2 × 6 analysis showed a significant main effect on “ISI” (*F*_4,26_ = 101.34, *P* < 0.001) but not on “time” (*F*_1,29_ = 0.03, *P* = 0.871) or a “time × ISI” interaction (*F*_4,26_ = 0.49, *P* = 0.787). For PAS this RM-ANOVA revealed both a significant “ISI” effect (*F*_4,26_ = 91.13, *P* < 0.001) and a significant “time” effect (*F*_1,29_ = 5.97, *P* = 0.021) but no “time × ISI” interaction (*F*_4,26_ = 1.65, *P* = 0.150).

A similar pattern was obtained in the “time” and “mean ISI” 2 × 3 RM-ANOVA. For anodal tDCS, the analysis showed a significant “mean ISI” effect (*F*_1,29_ = 128.02, *P* < 0.001) and no “time” effect (*F*_1,29_ = 1.01, *P* = 0.753) or “time × ISI” interaction (*F*_1,29_ = 1.06, *P* = 0.352). For PAS we found a significant “mean ISI” effect (*F*_1,29_ = 124.64, *P* < 0.001) and “time” effect (*F*_1,29_ = 7.768, *P* = 0.009) and a “time × ISI” interaction (*F*_1,29_ = 3.99, *P* = 0.024).

In the PAS experiments, subsequent dependent samples *t*-tests were conducted to compare paired-pulse measures before and after stimulation. These analyses showed an increase in all tested variables following PAS with significant differences for SICI at 2 ms ISI (*t*_1,29_ = 2.61, *P* = 0.014) and mean SICI (*t*_1,29_ = 2.42, *P* = 0.022) and the test pulse (*t*_1,29_ = 3.96, *P* < 0.001). Thus, the significant effects reported from the RM-ANOVA are very likely to be a consequence of the increase in the test pulse after stimulation. Due to the lacking time effect, no further *t*-tests were conducted for the anodal experiments.

### 4.5. Response Analysis

**Figure 3:**
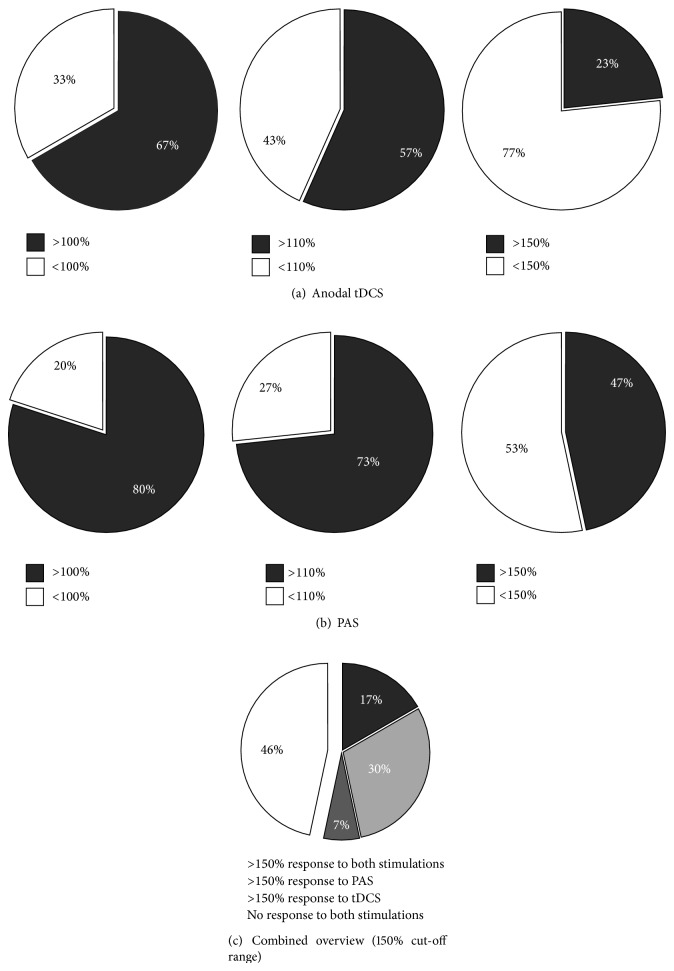
Individual response patterns of all subjects for (a) anodal tDCS and (b) PAS separated according to the defined cut-off ranges of >100%, >110%, and >150% relative to baseline MEP values (set as 100%). Responders (R) are depicted with grey coloured fields and nonresponders (NR) with white fields. (c) Grouped presentation of responders to both stimulation types (17%, dark grey), to PAS only (33%, light grey), or to anodal tDCS only (7%, intermediate grey) and nonresponders (46%, white) for the >150% cut-off range relative to baseline MEP values (set as 100%).

**Figure 4:**
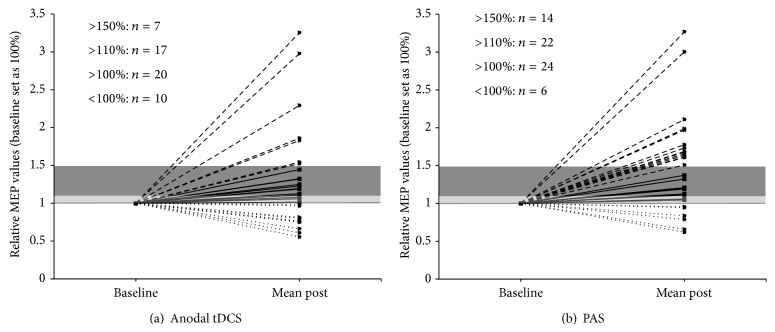
Presentation of the number of responders to (a) anodal tDCS and (b) PAS within the three different response ranges scaled in relative values, with 1 representing 100% of baseline MEP size. The >100% cut-off range is depicted in light grey and the >110% range in dark grey. Responders over 150% are shown above the dark grey bar and nonresponders (NR) underneath the black line representing 100% baseline MEP. Total numbers shown for each of the separate cut-off ranges.

To obtain an overview over the individual response patterns of all subjects three different response cut-offs were defined. These cut-offs defined response as an MEP size increase following the respective stimulation types over a cut-off of >100%, >110%, and 150% relative to the individual baseline (Figures 3 and 4).

Chi-Square (Chi^2^) tests were computed to compare the stimulation protocol and the respective individual response pattern. These analyses revealed trend-level differences between anodal and PAS responders at >110% (*P* = 0.052) and >150% (*P* = 0.058) cut-off ranges but not at >100% (0.243) in favour of the PAS stimulation.

Defining a decrease of SICI and an increase in ICF following LTP-protocols as response, we again defined three different cut-off ranges: >100%, >110%, and >150% increase of the respective relative mean values (post/pre: SICI_(2-3 ms)_; ICF_(9–12 ms)_). Chi^2^ tests were used to compare the distribution of responders between both experiments. For SICI decrease no significant difference in the distribution of responders was found (>100%: *P* = 1.000; >110%: *P* = 0.795; >150%: *P* = 1.000). In comparison, the analysis for ICF increase revealed a significant difference in the distribution of responders in all of the three ranges (>100%: *P* = 0.009; >110%: *P* = 0.002; >150%: *P* = 0.005) in favour of anodal tDCS.

In order to explore whether gender affected the MEP increase following stimulation, Chi^2^ tests were obtained from both experiments comparing distribution of response and gender. For all cut-off ranges, this analysis did not reveal any significant differences between gender and anodal tDCS (100%: *P* = 0.796; 110%: *P* = 0.491; 150%: *P* = 0.273) or PAS (100%: *P* = 0.855; 110%: *P* = 0.855; 150%: *P* = 0.261).

### 4.6. Correlational Analyses

Pearson correlation coefficients were used to examine the relationship between relative baseline values (age; standard deviation of MEPs; SICI 2 ms, SICI 3 ms, ICF 7 ms, ICF 9 ms, and ICF 12 ms) and the relative mean MEP values following stimulation in both experiments.

For PAS these analyses revealed a positive trend-level correlation between age and relative mean poststimulation MEPs (*r* = 0.345, *P* = 0.062), which was not observed after anodal tDCS (*r* = 0.010, *P* = 0.959). In addition, we observed for anodal tDCS a positive correlation between the relative ICF values at baseline (12 ms ISI) and the relative mean poststimulation MEP values (*r* = 0.557, *P* = 0.001). Concerning all other variables no significant correlations were observed (all *r* < 0.091; all *P* > 0.632).

To further investigate the impact of the observed ICF-correlation in the anodal experiments, we compared the relative baseline ICF values (12 ms) between responders and nonresponders in the anodal condition. These analyses revealed a trend-level difference in the case of the >100% cut-off range (*t*_1,28_ = 1.87, *P* = 0.072) but significantly higher relative baseline 12 ms ICF values for both >110% (*t*_1,28_ = 2.15, *P* = 0.041) and >150% (*t*_1,28_ = 3.55, *P* = 0.0014) in responders compared to nonresponders.

## 5. Discussion

The present results reveal a differential response pattern following two well-established LTP-protocols in a large sample of healthy controls. Both LTP-protocols (anodal tDCS and PAS25) induced a significant increase of MEPs for the observation period of 30 minutes. In the PAS25 experiments this MEP increase could be observed for nearly all poststimulation time points, whereas in the anodal tDCS experiments the increase of MEPs could only be shown when all time points were averaged. The likelihood to develop a meaningful MEP response was higher in the PAS25 compared to the anodal tDCS experiments, but the likelihood for a response in terms of an ICF-modulation showed the opposite pattern. In the anodal tDCS experiments, the baseline values for ICF at 12 ms showed a positive correlation with the increase of MEPs after stimulation and this value differed significantly between responders and nonresponders. In summary, our results indicate a sufficient increase of cortical excitability following anodal tDCS and PAS25 in terms but also demonstrate less individual variability than reported in previous studies.

### 5.1. Interindividual Response Differences

Considering the particular importance of interindividual response differences surprisingly limited data comparing NIBS protocols with different modes of action is available. One study compared the efficacy of three different LTP-protocols (intermittent theta-burst stimulation (TBS), anodal tDCS, and PAS25) in 56 healthy controls and the authors were not able to show significant effects on excitatory or inhibitory circuits when all subjects were analysed as a group [5]. This response pattern resembles the results of another study conducted on 18 subjects showing in the group-level analyses no increase in MEPs following intermittent TBS and PAS25 but a decrease in MEP amplitudes following continuous TBS [24]. For tDCS, a recently published study could not observe a modulation of MEP amplitudes following anodal or cathodal tDCS in 53 healthy subjects [4]. The fact that this study used an intensity of 2 mA for tDCS [4] whereas most tDCS studies used 1 mA [7, 25] should be taken into account. Recent evidence indicates that the increase in the tDCS intensity is not related to the efficacy of stimulation and that homeostatic mechanisms could counteract the efficacy of high stimulation intensity [26]. For TBS, one study showed in 56 healthy subjects that neither excitatory nor inhibitory TBS resulted in a change in MEP sizes after stimulation and that only 25% of all subjects showed the expected responses [2]. The lacking group-level response following different TBS protocols has now been reported from different groups (e.g., [27–30]). For PAS25, similar results with lacking increase in MEP amplitudes and a response rate in 14 out of 27 subjects were shown in one study [3]. This high response variability following standard PAS protocols was confirmed in other studies with limited sample sizes [31, 32].

The reason most frequently discussed for not being able to induce a general change in motor-cortical excitability in the presented studies with sufficient sample sizes is the high response variability across subjects [2, 4, 5]. Our findings deviate from the aforementioned reports. In our study, both plasticity protocols were effective on a group-level analysis and our results are in the range of the initial reports for PAS [14] and tDCS [10, 11, 17]. Furthermore, the likelihood to develop a MEP response was higher in the PAS25 experiments. Remarkably, only 23% responded to anodal tDCS, but 47% responded to PAS using the 150% cut-off. Comparing the mean MEP amplitudes after stimulation, a numeric but not statistical significant difference between the PAS25 and tDCS experiments could be observed. Thus, it may be assumed that PAS25 was more effective to increase MEP amplitudes. On the other hand, the likelihood for a response in ICF was higher in the anodal tDCS experiments. Furthermore, baseline ICF correlated positively with an increase in MEP amplitudes and MEP responders had significantly higher ICF baseline values compared to the nonresponders in the anodal experiment. It should be noted that a decrease in SICI and an increase in ICF have been reported following anodal tDCS [33] but not following PAS25 (for review see [1, 16]). Therefore, paired-pulse measures may not be suitable as outcome parameters for PAS25 but may be helpful to detect responders to anodal tDCS.

### 5.2. Correlation of Response and Baseline Excitability

In one study baseline SICI correlated with the PAS25 response [5] and another study showed that the PAS25 response correlated with SICI measured with a threshold tracking method [34]. Differences between these studies and our work may be the timing of the paired-pulse assessment and the configuration of the conditioning pulse. Our novel observation of an ICF-associated efficacy of anodal tDCS may allow hypothesizing that the after-effects of anodal tDCS appear to be located in facilitatory interneuron networks and may be dependent on synaptic modulation [35]. The reservation must be made that our design does not allow a mechanistic explanation of results due to the lack of pharmacological interventions.

We aimed to identify further baseline characteristics (other paired-pulse measures, age, gender, and variability in baseline MEPs expressed by the standard deviation) that may predict the response to anodal tDCS or PAS25, but no other factors were detected. We found a trend for a positive correlation between age and the MEP changes following PAS25. This is in contrast to previous reports of a negative correlation between age and the response to PAS [3]. However, our age range is outside the age range of an expected age-dependent decrease in cortical plasticity.

### 5.3. Limitations

We report the plasticity effects following NIBS in a single-session design and thus cannot rule out that repetitive sessions (as used in the clinical context) would have resulted in another distribution of plasticity. Furthermore, we focussed on two established LTP-protocols and as it is possible to modulate various parameters using tDCS (e.g., current intensity, current density) and PAS (e.g., ISI, individualised PAS, and target peripheral nerve) it may be possible that other configurations would have resulted in different response patterns. Regarding the paired-pulse measures, the lacking assessment of a paired-pulse response curve at different time points limits the generalizability of our SICI/ICF discussion. Furthermore, we did not readjust the test pulse intensity after the intervention in the paired-pulse paradigms. On the one hand evidence is available that the intensity of the test pulse affects the percent SICI/ICF [36], but on the other hand this is not found to be the case in the range of MEP sizes presented in our experiments [36, 37]. The MEP sizes following the test pulse of the paired-pulse paradigms were 1.04 ± 0.31 before and 1.23 ± 0.76 after anodal tDCS and, respectively, 1.11 ± 0.43 before and 1.64 ± 0.86 after PAS25. Further limitations are the lacking sham condition in our experiments and the fact that we did not use randomized ordered session of anodal tDCS and PAS25. Thus, we cannot rule out that the order of sessions had an impact on our results. For PAS, we used the ulnar nerve for our peripheral nerve stimulation and the FDI as target muscle. Most published PAS studies stimulated the median nerve and used the abductor pollicis brevis muscle as target (for review see [16]). However, different studies reported comparable changes in MEPs following both ulnar and median nerve stimulation, in the context of PAS protocols (for review see [16]). One important confounding factor could be attention. In our PAS experiment we asked the subjects to count the TMS pulse and the reported values are within a range (179.5 ± 1.5) [22, 23] indicating a sufficient level of attention in our experiments. During anodal tDCS no count of stimuli is conducted, possibly leading to different attention states compared with those in the PAS setup. Thus, the reported higher efficacy of PAS25 could also be led back to attention rather than to physiological differences between the protocols. Despite our large sample, the analyses of MEP response distribution did just barely miss the significance threshold. Thus, replication studies with larger sample sizes are needed to confirm some of our findings.

### 5.4. Summary and Conclusions

Our results show that two currently controversially discussed plasticity protocols are effective. However, in line with previous research, our sample also comprised a certain degree of nonresponders. The likelihood to be a nonresponder was dependent on the kind of stimulation protocol, on the defined thresholds and on the used target items. Our new observation that subject had a higher likelihood to be a responder using the high 150% MEP threshold in the PAS25 protocol may be explained by the fact that this protocol was more individualised (electrical and motor thresholds) and controlled for attention compared to anodal tDCS. Moreover, we were for the first time able to identify a new baseline parameter (ICF at 12 ms) that may predict the response to anodal tDCS. Future prospective studies need to independently confirm this parameter before it can be used as individual response predictor. It is reassuring that our response rates using the lowest threshold were much higher than those reported in other trials, but the response rates using the highest threshold are less optimistic.

## Figures and Tables

**Table 1:** Descriptive statistics of the data sample (data presented as mean ± standard deviation).

Variables	
Gender	f = 14 (47%); m = 16 (53%)
Age (years)	27.4 ± 4.8 (range 19–42)
Handedness	Right = 29 (94%); left = 1 (6%)
Body-height (cm)	176.5 ± 9.0
Smoking state	Nonsmoker = 17 (57%); smoker = 13 (43%)
Fagerstroem (score points)	3.0 ± 2.0

**Table 2:** Baseline comparisons of the dependent variables in both experimental setups. Data (untransformed) presented as mean ± standard deviation (SP: single-pulse measures; PP: paired-pulse measures).

Baseline values	Anodal tDCS	PAS	*P* value
RMT (%) SP	33 ± 6	33 ± 6	0.538
S1mV (%) SP	42 ± 8	42 ± 9	0.305
RMT (%) PP	42 ± 8	42 ± 8	0.540
S1mV (%) PP	52 ± 9	52 ± 10	0.815
1 mV MEP (mV)	1.074 ± 0.23	1.073 ± 0.26	0.953
2 ms SICI (mV)	0.445 ± 0.37	0.355 ± 0.27	0.129
3 ms SICI (mV)	0.371 ± 0.29	0.451 ± 0.50	0.570
7 ms ICF (mV)	1.371 ± 0.56	1.314 ± 0.74	0.571
9 ms ICF (mV)	1.589 ± 0.61	1.769 ± 0.92	0.469
12 ms ICF (mV)	1.637 ± 0.71	1.795 ± 0.93	0.560
I/O (90% RMT) (mV)	0.422 ± 0.03	0.053 ± 0.06	0.549
I/O (110% RMT) (mV)	0.489 ± 0.38	0.420 ± 0.37	0.300
I/O (130% RMT) (mV)	1.664 ± 0.97	1.946 ± 1.27	0.319
