# Corrigendum to “Efficacy and Interindividual Variability in Motor-Cortex Plasticity following Anodal tDCS and Paired-Associative Stimulation”

**DOI:** 10.1155/2015/903265

**Published:** 2015-09-02

**Authors:** Wolfgang Strube, Tilmann Bunse, Berend Malchow, Alkomiet Hasan

**Affiliations:** Department of Psychiatry and Psychotherapy, Ludwig Maximilian University, 80336 Munich, Germany

The authors wish to note that they discovered an error in the database of their paper “Efficacy and Interindividual Variability in Motor-Cortex Plasticity following Anodal tDCS and Paired-Associative Stimulation” [1] after publication in Neural Plasticity. Due to a data export error, one subject was included twice in the database resulting in the situation that one dataset was completely overwritten. The dataset of the other subject is unfortunately lost. They are glad to report that the subsequent necessary reduction of the sample size from 29 from 30 resulted in only subtle changes in all reported statistical values without any impact on the main significances reported. One analysis showed a significant result instead of the previously reported trend: “For PAS these analyses revealed a positive correlation between age and relative mean poststimulation MEPs (*r* = 0.369, *p* = 0.049).”

The complete (1) corrected results section (Section 4 in “Efficacy and Interindividual Variability in Motor-Cortex Plasticity following Anodal tDCS and Paired-Associative Stimulation”) and (2) the slight corrections in the tables and figures are as follows.

*Descriptive Statistics*. Subjects were aged between 19 and 42 years (mean 27.4 ± 4.9), 14 were female (48%), with one exception that all were right handed (*n* = 28, 97%), the average body-height was 176.0 ± 9 cm, and 13 were smokers (45%) with a mean Fagerstroem score of 3 (see Table 1). 

*Baseline Differences*. To compare baseline values in both experiments, paired-samples *t*-tests were computed for all depending variables: RMT, S1mV (both single- and double-pulse), 1 mV MEP, SICI (2 ms, 3 ms, mean 2-3 ms) and ICF (7 ms, 9 ms, and 12 ms, mean 9–12 ms), and recruitment curve (90%, 110%, and 130% RMT). None of the tested variables showed significant differences between the first and the second experimental session (all *p* > 0.212; see Table 2). 

*Excitability Changes over Time*. A repeated-measures analysis of variance (RM-ANOVA) was conducted with the factors “time course” (baseline, 0 min, 5 min, 10 min, 20 min, and 30 min) and “stimulation” (anodal tDCS, PAS). This analysis revealed a significant main effect on “time course” (*F*_5,140_ = 4.162, *p* = 0.001) but neither an effect on “stimulation” (*F*_1,28_ = 1.632, *p* = 0.212) nor on the “time course × stimulation” interaction (*F*_5,140_ = 0.621, *p* = 0.684). In addition, the overall RM-ANOVA with the factors “time” (baseline, mean post-MEPs averaged) and again “stimulation” (anodal tDCS, PAS) showed also a significant main effect on “time” (*F*_1,28_ = 11.016, *p* = 0.003) and no effect on “stimulation” (*F*_1,28_ = 1.036, *p* = 0.318) or a “time × stimulation” interaction (*F*_1,28_ = 1.485, *p* = 0.233).

RM-ANOVAs separately computed for both stimulation protocols showed a significant main effect on “time course” in the PAS-group (*F*_5,140_ = 3.760, *p* = 0.003) but not in the tDCS-group (*F*_5,140_ = 1.412, *p* = 0.224). To analyse the general excitability changes following both stimulation types, a mean value of all poststimulation time points was included into an additional RM-ANOVA analysis, which showed a significant main effect on “time” for both anodal tDCS (*F*_1,28_ = 4.267, *p* = 0.048) and PAS (*F*_1,28_ = 14.058, *p* = 0.001).

For anodal tDCS, paired-samples *t*-tests showed significant differences comparing baseline to the mean of all time points following stimulation (*t*_28_ = 2.07, *p* = 0.048). In the case of PAS, a significant increase in MEP size was found comparing baseline to the mean of all time points following stimulation (*t*_28_ = 3.75, *p* = 0.001) and at all single time points after stimulation (all *t*_28_ > 2.45, all *p* < 0.019) with the exception of 0 minutes (*t*_28_ = 1.64, *p* = 0.112) and 5 minutes after stimulation (*t*_28_ = 1.87, *p* = 0.072). Baseline MEPs did not differ between the anodal and the PAS condition (*t*_28_ = 0.03, *p* = 0.974) and also mean post-MEPs did not differ between the anodal and the PAS condition (*t*_28_ = 1.35, *p* = 0.187) (Figure 2).

To further explore the observed differences between the MEP increase in tDCS and PAS, we further conducted a RM-ANOVA of the standard deviations. This analysis revealed no significant effects on “time course” (*F*_5,140_ = 1.61, *p* = 0.161), on “stimulation” (*F*_1,28_ = 0.262, *p* = 0.613), and on “time course × stimulation” interaction (*F*_5,140_ = 1.02, *p* = 0.407). This finding can be explained by higher standard deviations after stimulation in both conditions.

A RM-ANOVA for the IO-curves with the factors “time” (baseline, after stimulation) and “intensity” (90%, 110%, and 130% RMT) revealed a significant main effect on “intensity” for both anodal tDCS (*F*_1,5;42,5_ = 165.95, *p* < 0.001) and PAS (*F*_1,5;42,1_ = 163.95, *p* < 0.001) but no effect on “time” (tDCS: *F*_1,28_ = 2.31, *p* = 0.140; PAS: *F*_1,28_ = 2.89, *p* = 0.100) and no “time × intensity” interaction (tDCS: *F*_2,56_ = 0.459, *p* = 0.634; PAS: *F*_2,56_ = 2.19, *p* = 0.121). 

*Paired-Pulse Measurements*. For paired-pulse measurements, two additional RM-ANOVAs were conducted for both anodal tDCS and PAS separately with the factors “time” (baseline, 15 min after stimulation) and all “ISI” (test pulse, 2 ms, 3 ms, 7 ms, 9 ms, and 12 ms) or “mean ISI” (test pulse, mean SICI (2 ms, 3 ms), and mean ICF (9 ms, 12 ms)). In the case of anodal tDCS, the 2 × 6 analysis showed a significant main effect on “ISI” (*F*_3,1;86,6_ = 101.03, *p* < 0.001) but not on “time” (*F*_1,28_ = 0.04, *p* = 0.854) or a “time × ISI” interaction (*F*_5,140_ = 0.58, *p* = 0.715). For PAS this RM-ANOVA revealed both a significant “ISI” effect (*F*_2,8;77,8_ = 85.99, *p* < 0.001) and a significant “time” effect (*F*_1,28_ = 5.31, *p* = 0.029) but no “time × ISI” interaction (*F*_5,140_ = 1.60, *p* = 0.164).

A similar pattern was obtained in the “time” and “mean ISI” 2 × 3 RM-ANOVA. For anodal tDCS, the analysis showed a significant “mean ISI” effect (*F*_2,56_ = 127.23, *p* < 0.001) and no “time” effect (*F*_1,28_ = 1.13, *p* = 0.740) or “time × ISI” interaction (*F*_2,56_ = 1.23, *p* = 0.299). For PAS we found a significant “mean ISI” effect (*F*_1,5;43,2_ = 117.90, *p* < 0.001) and a significant “time” effect (*F*_1,28_ = 6.90, *p* = 0.014) and a “time × ISI” interaction (*F*_2,56_ = 3.79, *p* = 0.029).

In the PAS experiments, subsequent dependent samples *t*-tests were conducted to compare paired-pulse measures before and after stimulation. These analyses showed an increase in all tested variables following PAS with significant differences for SICI at 2 ms ISI (*t*_28_ = 2.54, *p* = 0.017) and mean SICI (*t*_28_ = 1.70, *p* = 0.027) and the test pulse (*t*_28_ = 3.76, *p* < 0.001). Due to the lacking time effect, no further *t*-tests were conducted for the anodal experiments. 

*Response Analysis*. To obtain an overview over the individual response patterns of all subjects three different response cut-offs were defined. These cut-offs defined response as an MEP size increase following the respective stimulation types over a cut-off of >100%, >110%, and 150% relative to the individual baseline (Figures 3 and 4).

Chi-Square (Chi^2^) tests were computed to compare the stimulation protocol and the respective individual response pattern. These analyses revealed a significant difference between anodal tDCS and PAS responders at >110% (*p* = 0.050) and a trend-level difference between both protocols at >150% (*p* = 0.097) but not at >100% (*p* = 0.240) in favour of the PAS stimulation.

Defining a decrease of SICI and an increase in ICF following LTP-protocols as response, we again defined three different cut-off ranges: >100%, >110%, and >150% increase of the respective relative mean values (post/pre: SICI_(2-3 ms)_; ICF_(9–12 ms)_). Chi^2^ tests were used to compare the distribution of responders between both experiments. For SICI decrease no significant association was found for all of the three defined ranges (>100%: *p* = 0.788; >110%: *p* = 0.792; >150%: *p* = 1.000). In comparison, the analysis for ICF increase revealed a significant difference in the distribution of responders in all of the three ranges (>100%: *p* = 0.009; >110%: *p* = 0.001; >150%: *p* = 0.005) in favour of anodal tDCS.

In order to explore whether gender affected the MEP increase following stimulation, Chi^2^ tests were obtained from both experiments comparing distribution of response and gender. For all cut-off ranges, this analysis did not reveal any significant differences between gender and anodal tDCS (100%: *p* = 0.600; 110%: *p* = 0.434; 150%: *p* = 0.224) or PAS (100%: *p* = 0.639; 110%: *p* = 0.639; 150%: *p* = 0.239). 

*Correlational Analyses*. Pearson correlation coefficients were used to examine the relationship between relative baseline values (age; standard deviation of MEPs; SICI 2 ms, SICI 3 ms, ICF 7 ms, ICF 9 ms, and ICF 12 ms) and the relative mean MEP values following stimulation in both experiments.

For PAS these analyses revealed a positive correlation between age and relative mean poststimulation MEPs (*r* = 0.369, *p* = 0.049), which was not observed after anodal tDCS (*r* = 0.010, *p* = 0.958). In addition, we observed for anodal tDCS a positive correlation between the relative ICF values at baseline (12 ms ISI) and the relative mean poststimulation MEP values (*r* = 0.492, *p* = 0.007). Concerning all other variables no significant correlations were observed (all *r* < 0.051, all *p* > 0.074).

To further investigate the impact of the observed ICF-correlation in the anodal experiments, we compared the relative baseline ICF values (12 ms) between responders and nonresponders in the anodal condition. These analyses revealed a trend-level difference in the case of the >100% cut-off range (*t*_27_ = 1.85, *p* = 0.076) but significantly higher relative baseline 12 ms ICF values for both >110% (*t*_27_ = 2.13, *p* = 0.042) and >150% (*t*_27_ = 3.52, *p* = 0.002) in responders compared to nonresponders.[Table tab1] [Table tab2] [Fig fig1] [Fig fig2] [Fig fig3]

## Figures and Tables

**Figure 2 fig1:**
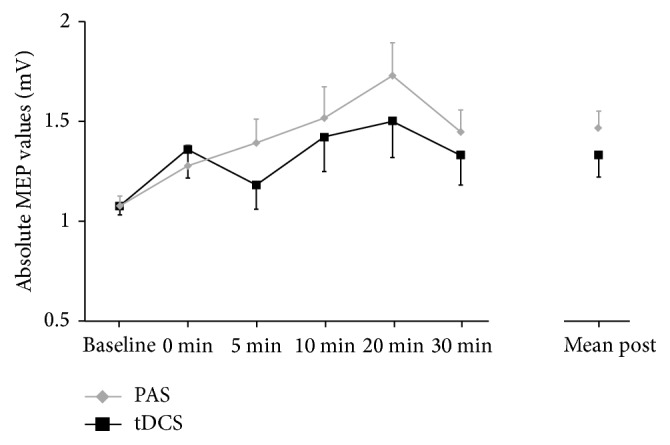
MEP values at baseline and all time points following anodal tDCS and PAS. MEP values are shown as untransformed values and scaled in mV and error bars representing the standard error of the mean.

**Figure 3 fig2:**
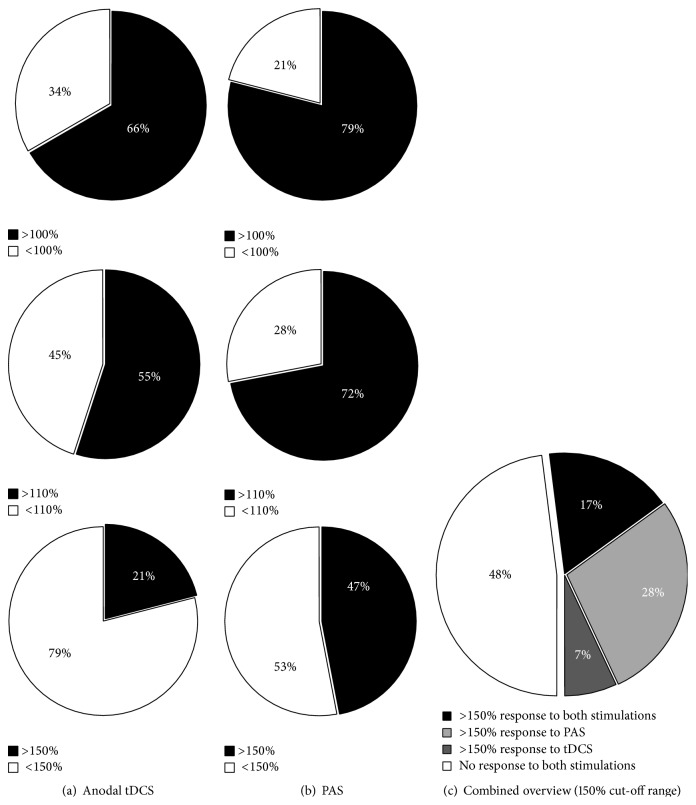
Individual response patterns of all subjects for (a) anodal tDCS and (b) PAS separated according to the defined cut-off ranges of >100%, >110%, and >150% relative to baseline MEP values (set as 100%). Responders (*R*) are depicted with grey coloured fields and nonresponders (NR) with white fields. (c) Grouped presentation of responders to both stimulation types (17%, dark grey), to PAS only (28%, light grey), or to anodal tDCS only (7%, intermediate grey) and nonresponders (48%, white) for the >150% cut-off range relative to baseline MEP values (set as 100%).

**Figure 4 fig3:**
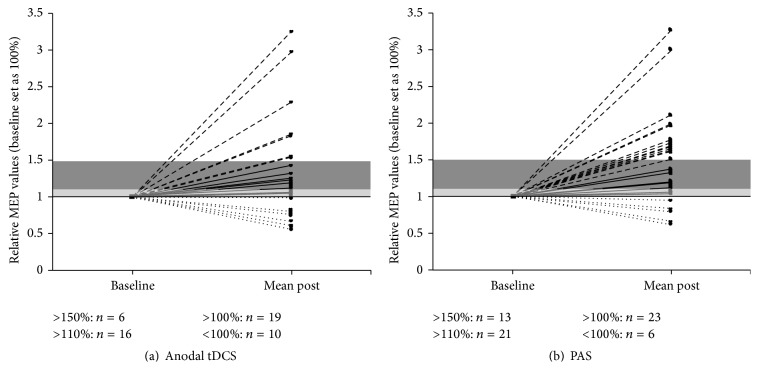
Presentation of the number of responders to (a) anodal tDCS and (b) PAS within the three different response ranges scaled in relative values, with 1 representing 100% of baseline MEP size. The >100% cut-off range is depicted in light grey and the >110% range in dark grey. Responders over 150% are shown above the dark grey bar and nonresponders (NR) underneath the black line representing 100% baseline MEP. Total numbers shown for each of the separate cut-off ranges.

**Table 1 tab1:** 

Variables	
Gender	f = 14 (48%); m = 15 (52%)
Age (years)	27.4 ± 4.9 (range 19–42)
Handedness	Right = 29 (97%); left = 1 (3%)
Body-height (cm)	176.0 ± 9.0
Smoking state	Nonsmoker = 16 (55%); smoker = 13 (45%)
Fagerstroem (score points)	3.0 ± 1.8

**Table 2 tab2:** 

Baseline values	Anodal tDCS	PAS	*p* value
RMT (%) SP	33 ± 6	33 ± 6	0.538
S1mV (%) SP	42 ± 8	42 ± 9	0.282
RMT (%) PP	42 ± 8	42 ± 8	0.498
S1mV (%) PP	52 ± 9	52 ± 10	0.815

1 mV MEP (mV)	1.074 ± 0.23	1.073 ± 0.26	0.958
2 ms SICI (mV)	0.445 ± 0.37	0.355 ± 0.27	0.211
3 ms SICI (mV)	0.371 ± 0.29	0.451 ± 0.50	0.436
7 ms ICF (mV)	1.371 ± 0.56	1.314 ± 0.74	0.547
9 ms ICF (mV)	1.589 ± 0.61	1.769 ± 0.92	0.498
12 ms ICF (mV)	1.637 ± 0.71	1.795 ± 0.93	0.560

I/O (90% RMT) (mV)	0.422 ± 0.03	0.053 ± 0.06	0.553
I/O (110% RMT) (mV)	0.489 ± 0.38	0.420 ± 0.37	0.360
I/O (130% RMT) (mV)	1.664 ± 0.97	1.946 ± 1.27	0.325
